# Genomic, Clinical, and Behavioral Characterization of 15q11.2 BP1-BP2 Deletion (Burnside-Butler) Syndrome in Five Families

**DOI:** 10.3390/ijms22041660

**Published:** 2021-02-07

**Authors:** Isaac Baldwin, Robin L. Shafer, Waheeda A. Hossain, Sumedha Gunewardena, Olivia J. Veatch, Matthew W. Mosconi, Merlin G. Butler

**Affiliations:** 1 Department of Psychiatry & Behavioral Sciences, University of Kansas Medical Center, 3901 Rainbow Blvd. MS 4015, Kansas City, KS 66160, USA; ibaldwin2@kumc.edu (I.B.); whossain@kumc.edu (W.A.H.); oveatch@kumc.edu (O.J.V.); 2 Department of Pediatrics, University of Kansas Medical Center, 3901 Rainbow Blvd. MS 4015, Kansas City, KS 66160, USA; 3 Schiefelbusch Institute for Life Span Studies and Kansas Center for Autism Research and Training, University of Kansas, Lawrence, KS 66045, USA; rshafer3@ku.edu (R.L.S.); mosconi@ku.edu (M.W.M.); 4 Department of Molecular and Integrative Physiology, University of Kansas Medical Center, Kansas City, KS 66160, USA; sgunewardena@kumc.edu; 5 Clinical Child Psychology Program, University of Kansas, Lawrence, KS 66045, USA

**Keywords:** 15q11.2 BP1-BP2 deletion, Burnside-Butler syndrome, clinical findings, cognition, neuropsychiatric behavior development, genomic characterization, exome sequencing, protein–protein interaction

## Abstract

The 15q11.2 BP1-BP2 deletion (Burnside-Butler) syndrome is emerging as the most common cytogenetic finding in patients with neurodevelopmental or autism spectrum disorders (ASD) presenting for microarray genetic testing. Clinical findings in Burnside-Butler syndrome include developmental and motor delays, congenital abnormalities, learning and behavioral problems, and abnormal brain findings. To better define symptom presentation, we performed comprehensive cognitive and behavioral testing, collected medical and family histories, and conducted clinical genetic evaluations. The 15q11.2 BP1-BP2 region includes the *TUBGCP5*, *CYFIP1*, *NIPA1*, and *NIPA2* genes. To determine if additional genomic variation outside of the 15q11.2 region influences expression of symptoms in Burnside-Butler syndrome, whole-exome sequencing was performed on the parents and affected children for the first time in five families with at least one parent and child with the 15q1l.2 BP1-BP2 deletion. In total, there were 453 genes with possibly damaging variants identified across all of the affected children. Of these, 99 genes had exclusively de novo variants and 107 had variants inherited exclusively from the parent without the deletion. There were three genes (*APBB1*, *GOLGA2*, and *MEOX1*) with de novo variants that encode proteins evidenced to interact with CYFIP1. In addition, one other gene of interest (*FAT3*) had variants inherited from the parent without the deletion and encoded a protein interacting with CYFIP1. The affected individuals commonly displayed a neurodevelopmental phenotype including ASD, speech delay, abnormal reflexes, and coordination issues along with craniofacial findings and orthopedic-related connective tissue problems. Of the 453 genes with variants, 35 were associated with ASD. On average, each affected child had variants in 6 distinct ASD-associated genes ($\bar{x}$ = 6.33, sd = 3.01). In addition, 32 genes with variants were included on clinical testing panels from Clinical Laboratory Improvement Amendments (CLIA) approved and accredited commercial laboratories reflecting other observed phenotypes. Notably, the dataset analyzed in this study was small and reported results will require validation in larger samples as well as functional follow-up. Regardless, we anticipate that results from our study will inform future research into the genetic factors influencing diverse symptoms in patients with Burnside-Butler syndrome, an emerging disorder with a neurodevelopmental behavioral phenotype.

## 1. Introduction

Chromosome 15 abnormalities have been reported for a number of years in the medical literature, specifically for Prader-Willi (PWS) and Angelman (AS) syndromes, the first examples of genomic imprinting in humans [1,2,3,4]. These disorders are generally due to a chromosome 15q11-q13 deletion depending on the parent-of-origin (i.e., PWS—paternal, AS—maternal). The typical 15q11-q13 deletions are classified as either Type I with deletions involving the proximal 15q breakpoint (BP1) and a distal 15q breakpoint (BP3), or Type II relating to a smaller 15q11-q13 deletion involving a second proximal breakpoint (BP2) and distal BP3. The larger Type I deletion is approximately 6 Mb and includes the *TUBGCP5*, *CYFIP1*, *NIPA1*, and *NIPA2* genes, while the smaller Type II deletion is approximately 5.5 Mb with all four genes intact [5].

Clinical and behavior differences have been reported for the past 15 years involving specific deletion classes in both PWS and AS. For example, individuals with PWS or AS having the Type I deletion, generally have more learning and behavioral problems compared to those with the Type II deletion [6,7]. Specifically, patients with PWS and larger deletions have more compulsions and maladaptive behaviors, as well as lower cognition, reading and math skills when compared to PWS patients with smaller deletions [3,5,6,8]. In AS, more impaired speech and seizure activity are noted in individuals with the larger deletion [4].

The emerging 15q11.2 BP1-BP2 microdeletion (Burnside-Butler) syndrome (BBS) encompasses the region between the PWS/AS chromosome 15q deletion breakpoints and includes the *TUBGCP5*, *CYFIP1*, *NIPA1*, and *NIPA2* genes. This microdeletion was consistently reported in early studies of patients presenting with unexplained behavioral, cognitive, and/or psychiatric problems [9,10,11]. Ho et al. [12] later summarized the results of ultra-high microarray single nucleotide polymorphism (SNP) analysis and found this microdeletion to be the most common cytogenetic finding observed in over 10,000 consecutive patients studied and presenting for genetic services with features of ASD or other neurodevelopmental disorders. Furthermore, a systematic literature review by Cox and Butler [10] found over 200 individuals reported with this microdeletion and grouped the clinical findings into five categories: (1) developmental, speech, and motor delays (73%, 67%, and 42% of cases, respectively); (2) dysmorphic ears and palatal anomalies (46%); (3) writing and reading impairment, memory problems, and verbal IQ scores ≤ 75 (50–60%); (4) general behavior problems, unspecified (55%); and (5) abnormal brain imaging, including a smaller brain surface with a thicker cortex (43%).

Notably, the four genes encoded in the 15q11.2 BP1-BP2 region are syntenic, bi-allelically conserved, and functionally predicted to interact with each other along with seven other genes (i.e., *IGFBP2*, *CFHR1*, *CFHR3*, *MNS1*, *SPG20*, *BMPR2*, and *SPAST*) recently reported and analyzed via in silico studies and STRING functional interactions network [13]. Rafi and Butler [13] also found that the encompassed four protein-coding genes showed 11 nodes and 34 edges. Network nodes represent proteins with splice isoforms or post-translational modifications collapsed into each node for all proteins produced by a single protein-coding gene. Edges represent protein–protein associations that jointly contribute to a shared function. These genes are at the center of our focus on genomics and clinical findings in individuals with the 15q11.2 BP1-BP2 deletion.

A major focus of our report is to identify variants if present in the non-deleted alleles of the four genes from the affected children in five unrelated families with the 15q11.2 BP1-BP2 microdeletion and characterize potential influences of genome-wide variation on symptom expression using whole-exome sequencing in trios. We assessed clinical, behavioral, and cognitive phenotypes as well as physical and motor development in relationship to genetic findings in each subject in separate families, the first study of its kind in this emerging syndrome.

## 2. Results

Five families were studied. Each family included one parent and at least one child with the 15q11.2 BP1-BP2 deletion, as confirmed by Methylation Specific-Multiplex Ligation Probe Amplification (MS-MLPA) assays. Each family was initially ascertained via the child having neurodevelopmental problems and/or ASD; chromosomal studies confirmed the 15q11.2 BP1-BP2 microdeletion. The parents were then cytogenetically analyzed to identify the deletion and five families with six affected children (three males and three females) were recruited for study.

### 2.1. Cognitive and Behavioral Features

Results of cognitive and behavioral testing obtained from members of five families with the 15q11.2 BP1-BP2 deletion are shown in Table 1 and Table 2. In terms of general cognitive functioning, 9/11 individuals with 15q11.2 BP1-BP2 microdeletions performed in the average to superior range; whereas, one affected child (Subject 3) scored just below the normal range for full-scale IQ, and one affected child (Subject 11) was unable to complete cognitive testing due to limited receptive and expressive language understanding and severe developmental delay. Academic abilities (spelling, reading comprehension, and math) were broadly commensurate with individuals’ cognitive abilities, though three individuals showed spelling abilities at least 1 standard deviation (SD) below their verbal abilities (Subjects 2, 3, and 9); one showed suppressed reading relative to verbal abilities (Subject 3), and one showed suppressed math relative to their nonverbal abilities (Subject 7). Verbal memory abilities appeared to be relatively intact among individuals. The majority of participants performed within 1 SD of the mean across subscales of the California Verbal Learning Test [14] (CVLT). Two participants (Subjects 1 and 3) scored below average on most subscales, however, and Subject 10 performed 1.5 SD below the mean on long delay recall and 3 SD below the mean on the recognition subscale. All individuals tested performed within the normal range or above on the Peabody Picture Vocabulary Test, Fourth Edition [15] (PPVT-4), indicating intact receptive language ability. Results from the Trail Making tests [16] indicated performance in the average to superior range across participants on Part A, demonstrating that psychomotor speed and planning was intact. In contrast, 4/10 participants showed performance on Part B that was greater than one 1 SD below their performance on Part A, suggesting the ability to rapidly and flexibly shift between response sets was selectively disrupted.

Results from Autism Diagnostic Observation Schedule, Second Edition [17,18] (ADOS-2) testing suggested 3/6 affected children met testing criteria for a classification of ASD (Subjects 2, 9, and 11; Subject 3 was 1 point below threshold). The Vineland Adaptive Behavior Scale, Third Edition [19] (VABS-III) indicated that all of the children in our cohort experienced deficits in a broad range of adaptive skills, with four of the six participants scoring two standard deviations below the population average on at least one subscale. For 6/7 affected children, rates and severity of repetitive behavior were comparable to children with ASD of similar ages [20]. The Broad Autism Phenotype Questionnaire [21] (BAP-Q) indicated autism-associated traits in 2/5 affected parents (Subjects 1 and 6) based on score thresholds defined by Sasson et al. [22]. Three of the five parents displayed repetitive behavior severity that was comparable to individuals with ASD of similar ages [20].

### 2.2. Sensorimotor Ability

Group means, standard deviations, and effect sizes for postural control are reported in Table 3. Six children with the 15q11.2 BP1-BP2 deletion, six age- and sex-matched control children, five affected parents, and four age- and sex-matched control adults completed sensorimotor testing. Children with the 15q11.2 BP1-BP2 showed medium to large elevations in center of pressure (COP) length (d = −0.64) and variability in medial-lateral (ML) sway relative to control children (d = −0.70), and small increases in variability of anterior-posterior (AP) sway (d = −0.38). Affected parents showed large increases in COP length relative to control adults (d = −0.94), and these differences were medium for variability of AP sway (d = −0.61).

### 2.3. Whole-Exome Sequencing

**Figure 1:**
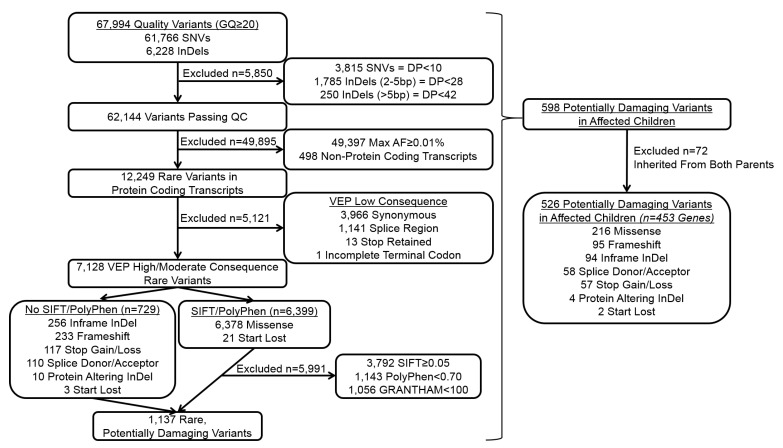
Quality control and variant selection. Overview of the criteria used to select high confidence, potentially damaging variants from whole-exome sequence data. Maximum allele frequencies (Max AF) were based on the maximum observed frequency across all reference populations available in the 1000 Genomes Project, the European Standard Population, and the Genome Aggregation Database. Abbreviations: GQ = genotype quality, SNVs=single nucleotide variant, InDels = insertion/deletion variant, DP = depth, VEP = Variant Effect Predictor.

**Figure 2:**
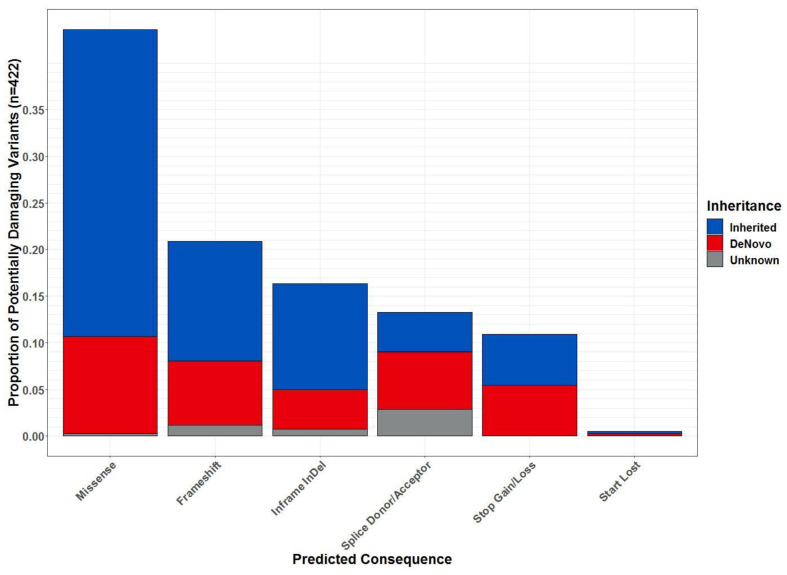
Predicted protein consequences of potentially damaging variants in affected children (Families A–D). Shown are the frequencies of Variant Effect Predictor consequences for variants identified in five affected children that were highly or moderately likely to damage the protein product. Data were not included for the affected child from Family E as the parents’ data were unavailable. Colors indicate variant consequences that were inherited (blue), de novo (crimson), or unknown (grey = uncharacterized Mendelian violations, or improbable homozygous de novo).

The per sequence Phred quality scale was above 35 for all the samples and 99.5% of sequenced reads mapped to the genome, resulting in ~37.1 million mapped reads per sample. There were 67,994 variants identified across the dataset. Of these, 526 were identified as high confidence, potentially damaging variants (PDVs) located in 453 distinct genes in affected children (see Figure 1). Each affected child had an average of 100 PDVs ($\bar{x}$ = 100.33, sd = 15.97) affecting 88 different genes ($\bar{x}$ = 88.00, sd = 12.88). The most prevalent type of PDV were missense variants (36.009%), followed by frameshifts (15.83%), inframe insertions and deletions (InDels; 15.67%), splice site variants (9.17%), losses or gains of stop codons (10.84%), protein altering variants (0.67%), and losses of start codons (0.33%; Figure 2). Almost all types of variants had a proportion that were inherited, de novo, or of unknown inheritance (Figure 2). In total, 132 of PDVs identified in affected children were predicted de novo. Some variants that were de novo in one child were observed inherited in others. Notably, there were 99 distinct genes with PDVs that were exclusively de novo. In addition, there were 125 PDVs in 107 genes that were inherited exclusively from the parent without the deletion.

**Figure 3:**
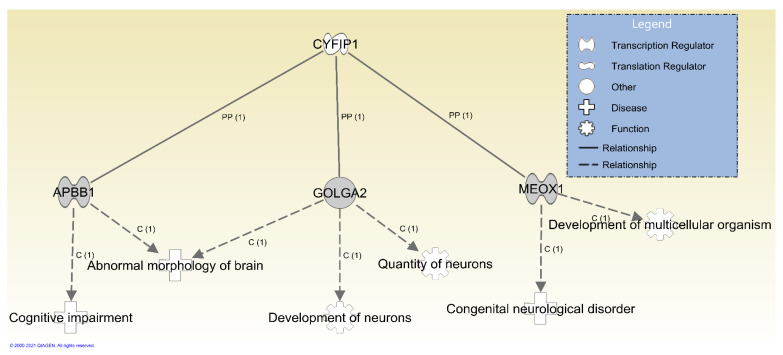
Genes with de novo variants with evidence for downstream relationships with *CYFIP1*. Shown are three genes prioritized following Ingenuity Pathway Analysis of all genes with de novo variants in affected children that were evidenced to be involved in the etiology of diseases relevant to symptoms observed in affected children. Included are protein–protein interactions (PP) and diseases and functions where variation in the gene is causal (C).

There were three genes with PDVs that were exclusively de novo evidenced to encode proteins that interact with the protein encoded by *CYFIP1* which is located in the region deleted in patients with BBS (Figure 3). These included an inframe deletion in *APBB1* in Subject 3, a missense mutation in *GOLGA2* in Subject 2, and a missense mutation in *MEOX1* in Subject 7. In particular, *APBB1* is associated with ASD (Supplemental Table S1). As noted above, Subject 3 was near the threshold for an ASD diagnosis based on the ADOS-2, and had a history of global developmental delay (Table 4). Notably, the location of the deletion identified in *APBB1* affects a region of the protein that contains simple sequence repeats (e.g., low-complexity region). As such, it is unclear whether or not this type of variation would influence phenotype expression.

**Figure 4:**
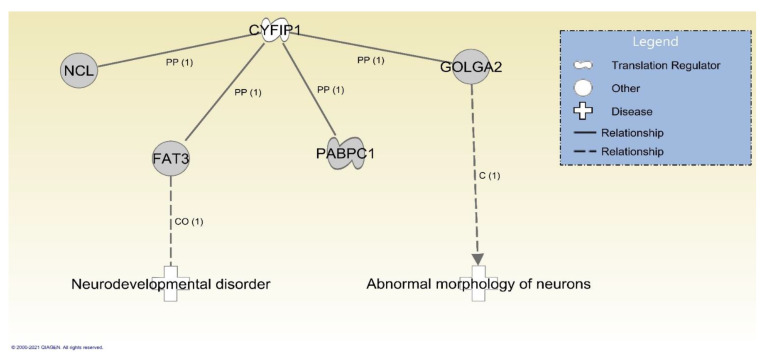
Genes with variants transmitted from non-deleted parents to affected children with evidence for downstream relationships with *CYFIP1*. Shown are genes prioritized following Ingenuity Pathway Analysis of all genes with variants in affected children that were inherited from parents without the 15q11.2 BP1-BP2 microdeletion that were also evidenced to be involved in the etiology of diseases relevant to symptoms observed in affected children. Included are protein–protein interactions (PP) and diseases and functions where variation in the gene is causal (C) or correlated (CO).

There were also genes with PDVs inherited exclusively from the parent without the 15q11.2 BP1-BP2 deletion that encoded proteins interacting with the products of two genes encoded in the deleted region, *CYFIP1* and *TUBGCP5* (Figure 4). Specifically, missense mutations were identified in two genes. These included *FAT3* where a PDV was observed in Subject 2; Subject 3 had the same variant in this gene and a variant in *GOLGA2*. Neither of these genes are associated with ASD or included on any of the evaluated clinical testing panels.

**Figure 5:**
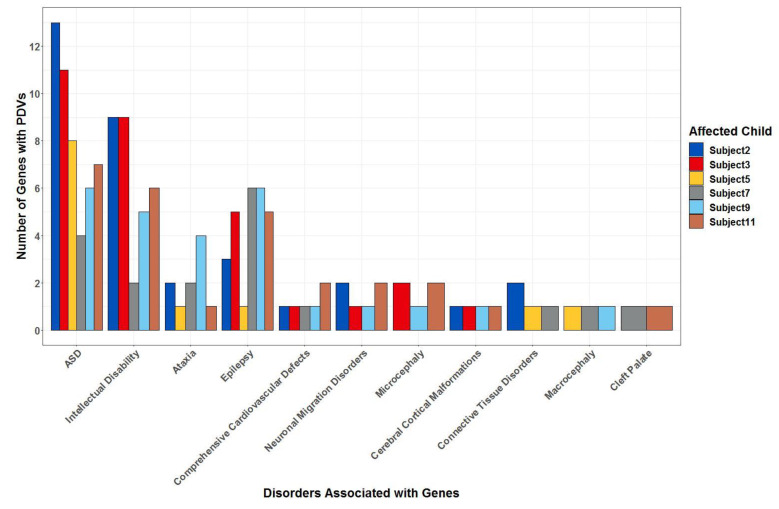
Number of genes with possibly damaging variants (PDVs) in each affected child associated with disorders of interest. Shown are the total number of genes with PDVs on the y-axis, that are also evidenced to be involved in the conditions on the x-axis. For autism spectrum disorder (ASD), evidence was based on the Simons Foundation for Autism Research Institute gene list. For other conditions, evidence was based on inclusion on clinical testing panels from Clinical Laboratory Improvement Amendments (CLIA) approved and accredited commercial laboratories.

Other genes of interest with PDVs were identified based on associations with clinical and physical findings in affected subjects as described in Table 1, Table 2, Table 4 and Table 5. Notably, an average of 17 genes ($\bar{x}$ = 17.3, sd = 5.3) with PDVs in each patient were associated with ASD. As seen in Figure 5, Subject 2 had the most ASD-associated genes with PDVs (*n* = 25). Compared to other affected children, Subject 2 also had the most severe repetitive behaviors measured via the Repetitive Behavior Scale – Revised [23] (RBS-R) and the most severe ADOS-2 score (Table 2). In addition, all affected children had PDVs in multiple genes that were included on clinical testing panels for intellectual disability ($\bar{x}$ = 8.7, sd = 4.7), ataxia ($\bar{x}$ = 6.8, sd = 2.9), epilepsy ($\bar{x}$= 6.2, sd = 2.8), comprehensive cardiovascular defects ($\bar{x}$ = 2.8, sd = 1.1), and neuronal migration disorders ($\bar{x}$ = 2.0, sd = 1.1). There were four subjects with PDVs in genes included on the cerebral cortical malformations panel, almost all of whom had some evidence of neurological issues (Table 4 and Table 5). Additionally, of note, both Subjects 7 and 11 had PDVs in genes included on the testing panel for cleft palate—*GLI2* and *FOXE1*, respectively— and had craniofacial malformations involving the mouth (Table 5). In addition, Subject 5 inherited a stop-loss variant in a connective tissue disorder gene, *FLCN*, from the parent with the deletion (Subject 4) both of whom had a history of musculoskeletal findings (Table 4). More details for all genes with PDVs meeting our inclusion criteria are provided in the Supplemental Table S1.

## 3. Discussion

This study is the first of its kind to characterize phenotypic, behavioral, and cognitive measures combined with exome sequencing in families with the 15q11.2 BP1-BP2 deletion. An initial goal was to determine if the sequences of one or more of the four genes in the 15q11.2 BP1-BP2 region showed a variant inherited from the parent with the intact (non-deleted) chromosome. When disturbed, the four genes in the 15q11.2 region are associated with cognitive impairment, speech and/or motor delay, dyslexia, and psychiatric/behavioral problems (e.g., attention deficit hyperactivity disorder (ADHD), autism, schizophrenia, or psychosis). The cardinal disease associations for the four contiguous genes in the 15q11.2 BP1-BP2 region are: *NIPA1*—Spastic Paraplegia 6; *NIPA2*—Angelman syndrome and Prader-Willi syndrome; *CYFIP1*—fragile X syndrome and autism; and *TUBGCP5*—Prader-Willi syndrome. The four genes are individually associated with PWS, ASD, schizophrenia, epilepsy, and Down syndrome. Collectively, all four genes have been associated with up to 75% of patients with ten distinctive neurodevelopmental disorders [13].

The addition of newly reported findings including ataxia, poor coordination, seizures, and congenital anomalies including palatal, heart, and ear defects along with structural brain disturbances [11] which are also associated with the four genes in the 15q11.2 BP1-BP2 region raises the question of whether these genes interact with other genes, their biological processes or molecular functions. These related genes may play a role in the clinical presentation causing core features of Prader-Willi and Angelman syndromes as additional clinical structural differences are seen in those with the four genes deleted in the typical 15q11-q13 Type I deletion seen in these syndromes. For example, dysfunctional variation in the *NIPA1* and *NIPA2* genes could impair the function of magnesium transport as both genes encode magnesium transporters [24,25]. Their biological processes and molecular functions could regulate axonogenesis and axon extension via relationships with bone morphogenetic protein (BMP) and signaling pathways, regulations of cellular and developmental growth, and interaction with the *FMR1* gene causing fragile X syndrome [13]; all pertinent and relevant to the reported variable clinical phenotypes seen in this microdeletion syndrome. We used whole-exome sequence data to identify other genes outside of the deleted region with possibly damaging variants to help detect genetic effects underlying expression of symptoms in the affected child bringing the family to medical attention. Detailed physical examinations and family pedigrees were performed, for the first time, by an experienced clinical geneticist trained as a dysmorphologist to characterize the phenotype and review of systems on each subject. In addition, cognitive and behavior testing, including motor assessments for ataxia or balance disturbances of each family member, were performed using various validated techniques and tests by experts in the field. These studies were the major outcome measures for comparison with the genomic data and analysis for similarities among our families with the 15q11.2 BP1-BP2 deletion.

### 3.1. Clinical and Neuropsychiatric Behavior Developmental Findings

We did not identify consistent patterns of cognitive impairments across individuals with 15q11.2 BP1-BP2 microdeletion syndrome. General cognitive, academic, and receptive vocabulary abilities were relatively intact with only one participant, Subject 11, not able to complete standardized IQ testing, showing indications of intellectual/developmental delay. Similarly, verbal memory abilities appeared to be unaffected across the majority of participants, though 3/10 participants showed mild deficits in suggesting that more selective issues in verbal memory and learning may impact a subset of individuals with the 15q11.2 BP1-BP2 microdeletion. Similarly, multiple participants (4/10) showed executive deficits characterized by a reduced ability to flexibly shift response sets. These participants were largely nonoverlapping with those showing verbal memory issues indicating that these cognitive effects may be relatively distinct across individuals with 15q11.2 BP1-BP2 deletion syndrome. Several children had a history of learning problems reported by parents, school records, or neuropsychological evaluations.

Our results suggest that individuals with 15q11.2 BP1-BP2 deletions have increased risk for ASD. The prevalence of ASD in the general population is estimated to be 1 in 54 (1.8%) [26]; however, in our sample, we found that 3/6 (50%) of affected children met testing standards for a diagnosis of ASD and 2/5 (40%) of affected parents demonstrated elevated autistic traits (one additional child, Subject 3, and one additional parent, Subject 10, scored just below the cutoff for the ADOS-2 and BAP-Q, respectively). Consistent with high rates of ASD and ASD-related traits in our sample, we observed high rates of repetitive behavior in a majority of our participants with 5/6 affected children and 3/5 adults demonstrating severity of repetitive behavior that is comparable to similarly aged persons with ASD [20]. Of note, our sample of 15q11.2 BP1-BP2 deletion carriers (parents) may actually underrepresent the prevalence of ASD in the population of these carriers since persons who are parents and those who volunteer to participate in research and travel significant distances are likely to represent a cohort with relatively mild symptoms. Notably, all of the affected children were observed to have variants in multiple genes that were associated with ASD or found on the intellectual disability testing panel.

One limitation to classification of ASD in this sample is the use of only one observational diagnostic tool (the ADOS-2), rather than using a combination of clinical observation, parent interviews and strict DSM-V criteria to confirm diagnosis; however, scores on the ADOS-2 combined with scores on the Vineland and RBS-R strongly indicate elevated rates of ASD and ASD-related traits in our sample. Similar to findings from studies of individuals with idiopathic ASD, the majority of our participants (9/11) showed adaptive functioning abilities below the mean for their age. These findings suggest that the 15q11.2 BP1-BP2 deletion confers increased risk for ASD and functional impairments independent of selective impacts on cognitive abilities.

Most of the individuals in this sample presented with at least one dysmorphic feature, some of which were present across multiple families. Five had abnormal ear findings such as broad, soft, fleshy, or overfolded ears. The child of Family C also had a smooth upper lip and philtrum. In the case of Family E, both the father and child had a broad, round face as well as broad hands. Flat feet were present in three individuals. Both the mother and child of Family B had small upper incisors. Six of the participants had eye findings demonstrating ptosis including both affected individuals from Family C and the father of Family E. The connective tissue finding of mild scoliosis was observed in two unrelated individuals whereas kyphosis was found in one other participant. Hyperextensibility or instability of various joints was a common feature, with unrelated individuals demonstrating a positive Beighton hyperflexibility score of at least six out of nine showing hypermobile joints. The 6-year-old child of Family A had a history of ankle instability and wore leg braces. Leg asymmetry was also found in two unrelated individuals. Pectus carinatum was seen in one individual while four participants had loose, soft skin, and two unrelated individuals had birth marks. Two unrelated affected children had a reported history of delayed wound healing.

Neurological problems were also present in several children. Two were diagnosed with epilepsy, one had non-essential tremor, and the child of Family D had gross hypotonia. The mother and child of Family D were both found to have decreased deep tendon reflexes, whereas the child of Family B had increased deep tendon reflexes. Ataxia was seen in the child of Family E. Motor delay was also relatively common, with four affected children showing delayed motor milestones. Motor deficits in affected individuals were also evident in our tests of postural control. While this test does not have normed scores or clinical cutoffs, group averages and effect sizes indicate that both children and adults with the 15q11.2 BP1-BP2 microdeletion show increased variability of postural sway relative to non-affected controls. This is consistent with findings of increased variability of motor behavior in neurodevelopmental disorders including Prader-Willi syndrome [27], ASD [28,29], and fragile-X associated disorders [30], and involvement of ataxia-related genes in 15q11.2 BP1-BP2. Of note, all affected children had PDVs in genes evidenced to cause ataxia, epilepsy, comprehensive cardiovascular defects, and neuronal migration disorders. In addition, many children had PDVs in genes involved in connective tissue disorders, cerebral cortical malformations, micro/macrocephaly, and congenital malformations with craniofacial defects that are included on the clinical cleft palate DNA testing panel.

### 3.2. Protein–Protein Interactions and Functions Related to NIPA1, NIPA2, CYFIP1 and TUBGCP5 Genes in the 15q11.2 BP1-BP2 Region

Of particular interest are the genes that had either a de novo variant, or a variant inherited from the parent without the deletion that encode proteins that interact with products of the four genes in the 15q11.2 BP1-BP2 region. As reported by Rafi and Butler [13] when examining the protein–protein interactions of the four genes in the 15q11.2 BP1-BP2 region, the predicted biological processes can be summarized as follows: regulation of cell growth, magnesium ion transmembrane transport, regulation of axonogenesis, regulation of plasma membrane bounded cell projection organization, positive regulation of axon extension, regulation of cellular response to growth factor stimulus, regulation of developmental growth, positive regulation of cell projection organization, mitotic spindle organization, regulation of BMP signaling pathway, and positive regulation of plasma membrane bounded cell projection assembly.

Notably, NIPA1 protein was observed in our previous study to interact with 11 other proteins. Five (45%) of the 11 proteins were members of the BMP superfamily, three (27%) were BMP receptors and TGFB1 (9%) protein, indicating that three-fourths of the NIPA1 interacting proteins are important for developmental bone morphogenesis or multifunctional proteins that control proliferation, differentiation, and other functions in many cell types. The NIPA2 protein interacted with 19 other proteins with three (16%) involved with the BMP protein superfamily, three (16%) proteins interact with BMP receptors, ACVR1, TGFBR1, and six members of the SMAD superfamily of proteins (42%); all playing a role as intracellular signal transducers and transcriptional modulators activated by TGFB, thereby impacting bone morphogenesis and its related functions. Specifically, the Spastin protein, encoded by *SPAST,* was observed to interact with both NIPA1 and NIPA2. A variant in *SPAST* was found in Subject 9. This child had hypotonia and history of fine and gross motor delay as well as autism. Spastin severs polyglutamylated microtubules and likely has a role in axon growth and branching [31,32]. Mutations in both *SPAST* and *NIPA1* have been identified as causes of hereditary spastic paraplegia, a condition which causes progressive weakness and spasticity of the legs [33,34]. De novo variants in *SPAST* are evidenced to be associated with ASD with comorbid spastic paraplegia [35].

The CYFIP1 protein is also reported to interact with other proteins having a wide range of activity with functions related to cytoskeleton organization and actin filament binding with cell-matrix adhesion, MAP kinase signal transduction of cell growth, survival and differentiation, stimulation of glucose uptake, intracellular protein breakdown and tissue remodeling with mediation of translational repression [36]. We observed that five additional genes with either a de novo or non-deleted parent inherited variant in the affected child encode proteins that interact with CYFIP1. *GOLGA2* encodes a protein that acts as a membrane skeleton that maintains the structure of the Golgi apparatus. Mouse models of this gene indicate its involvement in brain morphology and the development and quantity of neurons [37,38]. Loss of this gene also resulted in ataxia in mice [37]. Subject 2 had a PDV in this gene as well as a disturbed motor phenotype, ASD, and neuropsychiatric behavior developmental phenotypes. *MEOX1* encodes a mesodermal transcription factor that plays a key role in somitogenesis, specifically sclerotome development. *MEOX1* is involved in overall organism development in humans [39] and mutations in mice result in evidence of congenital neurological disorders [40]. Subject 7 had a de novo PDV in *MEOX1* along with connective tissue defects, congenital malformations, epilepsy, and neuropsychiatric behavior developmental phenotypes. Finally, *FAT3* encodes an atypical cadherin protein and may play a role in the interactions between neurites derived from specific subsets of neurons during development. While *FAT3* was not included in any of the disease association categories we directly evaluated, missense mutations in this gene are associated with the neurodevelopmental disorder, Hirschsprung disease (https://www.ncbi.nlm.nih.gov/clinvar/RCV000201304.1/). Both Subjects 2 and 3 had a PDV in this gene and evidence of a neurodevelopmental phenotype and should be monitored for gastrointestinal issues.

### 3.3. Identified Gene Variants with Potential Clinical Significance

As the first effort to identify variants in the four 15q11.2 BP1- BP2 genes in this microdeletion syndrome, we analyzed the non-deleted alleles in affected patients and assessed family members as well, using whole-exome sequencing in order to compare the genomic data of related genes with clinical, cognitive, and behavioral data. Some of these variants are identified by the gene panels as potentially contributing to multiple phenotypes in our subjects, as in the case of *MLC1*, which was a candidate for macrocephaly and motor delay in Subject 9 and for contribution to epilepsy in Subject 7. All variants passing inclusion criteria for possibly damaging the gene in which it is located, and the corresponding evidence for clinical significance of having a variant in the gene, can be found in Supplemental Table S1.

Other particular genes of interest with PDVs include numerous genes of the collagen or COL group which code for proteins that make up various subtypes of collagen. Disturbances in these genes are known to cause several connective tissue disorders [41]. For example, in addition to the stop-loss variant in the connective tissue disorder gene *FLCN*, Subject 5—who had significant joint hyperextensibility—inherited a frameshift in *COL5A3* from the parent with the deletion. Additional missense variants in collagen-encoding genes, *COL21A1* and *COL6A2*, were inherited from the non-deleted parent in Subject 7, who had joint hyperflexibility. Specifically, variation in *COL6A2* is associated with Ullrich congenital muscular dystrophy 1 (https://omim.org/entry/254090) (accessed on 6 February 2021). Subject 9 had an inframe deletion in *COL4A3* and a missense variant in *COL6A6* that was inherited from the deleted parent. While no joint hyperflexibility was noted, this patient had flat feet which may reflect collapse of connective tissues of the midfoot. Dysfunction in collagen proteins may also manifest as other symptoms. *COL4A3*, for instance, is implicated in disorders resulting in renal failure (https://omim.org/entry/120070) (accessed on 6 February 2021) and Subject 9 was also noted to have a history of constipation. Furthermore, variation in *COL6A6* has been implicated in skin disorders [42]. Subject 11, also without joint hyperflexibility, had the same missense variant identified in *COL6A6* and had birthmarks noted on the thigh and forehead.

Finally, *CDK19* gene is another candidate explaining clinical findings in Subject 11 who had a de novo missense variant in this gene. A disturbance in this gene has been associated with bilateral congenital retinal folds, microcephaly, and intellectual disability [43]. Of these findings, intellectual disability was present in Subject 11.

## 4. Materials and Methods

### 4.1. Families with 15q11.2 BP1-BP2 Deletion or Burnside-Butler Syndrome (BBS)

A total of five families with an affected child diagnosed with the 15q11.2 BP1-BP2 microdeletion were recruited and extensively evaluated using a series of cognitive, behavioral and motor assessments, family and medical histories, physical examination, and exome sequencing analyses. All participants or their legal guardians signed informed consent forms approved by the Institutional Review Board at the University of Kansas Medical Center (KUMC) before entry into the study. The five families included six affected children (3 males and 3 females) with one family having two affected children (Family A, Subjects 2 and 3) with BBS. Four mothers and one father had a confirmed 15q 11.2 BP1-BP2 deletion. Methylation-specific multiplex ligation-dependent probe amplification (MS-MLPA) was performed on two families for confirmation of the parents’ cytogenetic diagnoses using existing methodology [36].

### 4.2. Cognitive and Behavioral Measures

Cognitive and behavioral assessments were administered to all members of our study cohort (except where otherwise noted). All interviews and observational measures were conducted by trained members of our study team. The following cognitive and behavioral measures were administered:

The Wechsler Abbreviated Scales of Intelligence [44] (WASI-II) was used to assess general cognitive abilities including verbal, perceptual (nonverbal), and full-scale IQ.

The Wide Range Achievement Test-4 [45] (WRAT-4) was used to assess basic academic skills implicated previously in BBS, including sentence comprehension, word reading, spelling, and math computation.

The California Verbal Learning Test [14] (CVLT) is a comprehensive assessment of verbal learning and memory that specifically measures short delay free recall, short delay cued recall, long delay free recall, long delay cued recall, and long delay recognition. The CVLT-II was used in adolescents and adults (aged 16 to 89 years; N = 6), while the California Verbal Learning Test for Children (CVLT-C) was administered to children (aged 5 to 16 years; N = 4).

The Peabody Picture Vocabulary Test, Fourth Edition [15] (PPVT-4) was used to assess receptive vocabulary. During the PPVT-4, participants are instructed to identify the picture that best matches a target word. The number of correctly identified pictures was examined.

The Trail Making Test [16] is a commonly used assessment of visual search, processing speed, and cognitive flexibility consisting of two parts: Part A primarily measures visual search and processing speed as participants draw lines connecting numbers in sequential order as fast as possible. Part B assesses cognitive flexibility as participants must alternate between connecting letters and numbers as fast as possible (A-1-B-2-C-3, etc.). Reaction time and the number of errors made were examined for Parts A and B.

The Autism Diagnostic Observation Schedule, Second Edition [17,18] (ADOS-2) is a semi-structured play- and conversation-based assessment of the core social, communication, and repetitive behaviors in ASD. Module 3 (for children/adolescents with fluent speech) was used for all children in our study cohort except one non-verbal child, who as noted in Table 2, was administered Module 1 (for children 31 months or older who are preverbal or only use single words). The social-affective and total algorithm scores were examined. Higher scores reflect more severe symptoms of ASD.

Broad Autism Phenotype Questionnaire [21] (BAP-Q) measures traits relating to the broad autism phenotype—the presence of core diagnostic symptoms of ASD (e.g., social/communication impairments and restricted, repetitive behaviors) that occur below the threshold for a clinical diagnosis. This questionnaire consists of three subscales: social abnormalities, pragmatic language difficulties, and rigid personality and a desire for sameness. The BAP-Q was administered only to the parents in our study cohort. We used cutoff scores defined by Sasson et al. [22] (Males ≥ 3.55, Females ≥ 3.17), where higher scores indicate greater presence of ASD traits.

Vineland Adaptive Behavior Scales-Third Edition [19] (VABS-III) is a series of semi-structured caretaker interviews assessing current adaptive and daily living skills across four domains: communication, daily living skills, socialization, and motor skills.

The Repetitive Behavior Scale—Revised [23] (RBS-R) is a rating scale that assesses five categories of repetitive behavior (motor stereotypy, repetitive self-injury, compulsions, routines/sameness, restricted interests) commonly observed in individuals with developmental disabilities. Adult participants provided self-reported responses to the RBS-R, and a parent or caregiver provided responses on behalf of the child participants. Higher scores indicate greater severity of repetitive behavior. This measure does not have defined clinical thresholds, so we compared scores to age-dependent averages from a large cohort study of individuals with ASD [20].

### 4.3. Clinical Evaluation and Physical Examinations

A complete physical examination was performed by a clinical geneticist (MGB) with anthropometric measures (e.g., head circumference, ear length, inner and outer canthal distances, hand and mid-finger lengths) and data recorded including a three-generation family pedigree. Past medical histories and review of systems were included for cognition, behavior, seizures, pulmonary, cardiac, gastrointestinal, genitourinary, renal, musculoskeletal, cutaneous, immune, and hematology. Clinical photographs were obtained following written consent.

### 4.4. Postural Control Testing

Participants completed a postural control task to assess gross sensorimotor ability. Data for children and adult participants with the 15q11.2 BP1-BP2 deletion were compared to age- and sex-matched control participants who were unrelated to the families affected by the BBS deletion. Control participants completed postural control testing as part of larger studies taking place at two separate research sites. Where noted, some of the task parameters differed between these two sites.

Postural control was assessed using an AMTI (American Mechanical Technology, Inc., Watertown, MA, USA) AccuGait strain gauge force platform. Participants were tested bare footed in a well-lit room. They were instructed to stand as still as possible on the platform with their feet shoulder width apart and their arms resting at their sides. Participants completed three trials each lasting 30–45 s.

To standardize the duration of data analyzed, the first five seconds of each trial were removed and only the subsequent 20 s were included for analysis. Trials during which the participant lost balance or did not remain still for the duration of the trial (e.g., took a step, sneezed, spoke, walked away, etc.) were excluded from analysis. Participants who had fewer than two useable trials were excluded from analyses.

The center of pressure (COP) time series were derived from the force and moment data during standing posture. The time series of each trial was down-sampled to 100 Hz (for five control participants, data were down-sampled to 200 Hz) and low-pass filtered using a fourth-order double pass Butterworth filter with a cutoff frequency of 6 Hz.

The variability of each participant’s postural sway was quantified using the standard deviation (SD) of the COP in both the medial-lateral (ML) and anterior-posterior (AP) directions, as has been done in previous studies [30]. SD values were log transformed to correct for skewed distributions.

Data analyses were conducted separately for adults and children. Due to the small sample size of this study, we limited our analyses to the calculation of group means, standard deviations, and effect sizes (Cohen’s d) and interpreted as small (d = 0.2), medium (d = 0.5), or large (d = 0.8) according to Cohen’s standards [46]. One child with the 15q11.2 BP1-BP2 deletion (Subject 11) did not complete any postural control testing, so data from this participant and the matched control were not included in the analyses.

### 4.5. DNA Extraction

Saliva and buccal cells were collected using a Saliva DNA Collection and Preservation Device (Norgen Biotek Corporation, Thorold, Ontario, CA, USA). Genomic DNA was isolated and purified using a Saliva DNA Isolation Kit (Norgen Biotek Corporation) according to manufacturer’s protocol.

### 4.6. Methylation Specific-Multiplex Ligation Probe Amplification (MS-MLPA)

The MS-MLPA assay is a standard laboratory assay to examine for chromosome 15 deletions and was used to identify the presence or absence of the 15q11.2 BP1-BP2 deletion prior to enrolling individuals in this study. MS-MLPA was performed using reagents and kits obtained from MRC-Holland (Amsterdam, The Netherlands) including ME028-C1 kits containing sequence specific probes along the length of the 15q11.2-q13 region. The C1 kit contains 47 MLPA probes for copy number detection using fragment analysis following manufacturer’s instructions and reported elsewhere [36].

### 4.7. Whole-Exome Sequencing

Genomic DNA (5 μg) samples from four families (5 affected children and 4 sets of parents) were exome sequenced at the KUMC Genomics Core facility, and one family was sequenced in a commercial laboratory (GeneDx, Gaithersburg, MD, USA). Exome sequencing was performed using the TruSeq Exome Library Prep Kit (Illumina FC-150-1001, San Diego, CA, USA). High molecular weight (HMW) genomic DNA (gDNA) was fragmented using the Covaris S2 Ultra-Sonication system. Following fragmentation, DNA was end-repaired and 3’ adenylated prior to adaptor ligation using TruSeq DNA indexed adapters. The DNA libraries were equalized to 233 or 250 ng and pooled for two rounds of enrichment using the TruSeq Exome capture probes. TruSeq Exome capture probes target 45 Mb of coding sequence from >98% of RefSeq, Consensus CDS (CCDS), and Ensembl coding content. Following the final exome capture, the enriched libraries were amplified using polymerase chain reaction (PCR) to generate sufficient yield for sequencing. Enriched amplified libraries were validated using the Agilent Bioanalyzer 2100 system and quantitated using the Roche LightCycler96 RealTime PCR system. Final library concentration results were used to dilute the library pool to 2 nM and pooled for multiplexed sequencing on a NovaSeq 6000 sequencing machine (Illumina, San Diego, CA, USA). The onboard clonal clustering procedure was automated during the NovaSeq 6000 sequencing run. The 100-cycle paired end sequencing was performed using the NovaSeq 6000 S2 Reagent Kit—200 cycle (Illumina 20012861). Following sequencing, the raw base call files (.bcl) were converted to fastq files and de-multiplexed into individual libraries using Illumina’s bcl2fastq2 software.

### 4.8. Variant Calling and Quality Control Procedures

The initial read quality was assessed using the FastQC software1 [47]. The sequenced reads were then aligned to the human genome (hg38) using the Burrows–Wheeler aligner (BWA) [48]. The initial read quality was assessed using the FastQC software1 [47]. The sequenced reads were then aligned to the human genome (hg38) using the BWA [48]. Variant analyses of these data were performed in accordance with the Genome Analysis Toolkit (GATK) variant calling best practices pipeline [49]. Variant calling was as follows: The “MergeBamAlignment” tool was used to incorporate metadata to the aligned BAM files. The “MarkDuplicates” tool was used to locate and tag duplicate reads in the BAM files. The “BaseRecalibrator” tool was used to generate a recalibration table for base quality score recalibration (BQSR). Known polymorphic sites obtained from the GATK resource bundle were provided to this tool (Mills and 1000 G gold standard insertions or deletions (InDels) hg38 vcf, Homo sapiens assembly38 dbsnp138 vcf). The “ApplyBQSR” tool was run next in the two-stage process of base quality score recalibration. SNPs and InDels were called using the “HaplotypeCaller” tool. The resulting gVCF files were combined using the “CombineGVCFs” tool to form a multi-sample gVCF file. This file was passed for Joint genotyping using the “GenotypeGVCFs” tool. A recalibration model to score variant quality was built separately for InDels and SNPs using the “VariantRecalibrator” tool. The tool “ApplyVQSR” was then used with these models in the second stage of the variant quality score recalibration (VQSR) process to filter the input variants based on the recalibration table. The resulting data were further processed using the Genotype Refinement Workflow with the goal of improving the accuracy of genotype calls and discarding unreliable genotype calls. The first step of this process was to calculate the genotype posterior probabilities given family and known population genotypes using the “CalculateGenotypePosteriors” tool. The pedigree information of the families and a high confidence SNP file was provided to this tool. Variant calls were hard filtered to exclude genotype quality scores less than 20 (GQ < 20.0).

Figure 1 shows additional quality control and variant selection details from the whole-exome sequencing analysis. Specifically, single nucleotide variants (SNVs) were required to have read depth ≥10, InDels were required to have depth ≥28X when 2–5 base pairs (bp) long and ≥42X when 5–200 bps. To identify SNVs and InDels passing all quality control that were potentially damaging, the functional effects of variants on encoded proteins were predicted using the “SnpEff [50]” and Ensembl Variant Effect Predictor (VEP) tools [51]. Rare variants—with a maximum allele frequency (AF) <0.01% based on reference populations available in the 1000 Genomes Project, the European Standard Population and the Genome Aggregation Database—located in protein coding gene transcripts were selected. Rare variants predicted by VEP to have consequences that were moderately (i.e., inframe InDels, missense, or protein altering) or highly likely (i.e., splice site alterations, gains or losses of stop codons, loss of start codons, or frameshifts) to damage the protein products were then prioritized. Missense and start loss SNVs were further evaluated for potential deleterious effects using Sorting Intolerant From Tolerant (SIFT) [52], PolyPhen [53], and Grantham substitution scores [54] with inclusion criteria as follows: SIFT score < 0.05, PolyPhen score ≥ 0.70, and Grantham score ≥ 100 [54]. We then prioritized variants in affected children that were predicted de novo, or inherited from only one parent as additional evidence for potential clinical relevance.

### 4.9. Functional and Clinical Characterization of Genes with Variants

The implicated diseases and biological functions of the genes with variants of interest were then identified using the Ingenuity Systems Pathway Analysis (IPA) tool [55]. Variant effects analyses were run considering molecules and/or direct relationships that were experimentally observed in mammals (i.e., humans, mice, rats) from all data sources. Two separate analyses were run on genes with de novo variants and genes with inherited variants. As it was expected that de novo variants were more likely to represent clinically relevant findings [56], variant effects analysis of genes with de novo PDVs were conducted using evidence from all possible mutation consequences, including unclassified and silent mutations. For genes with PDVs that were transmitted to affected children from the parent without the 15q11.2 BP1-BP2 microdeletion, only evidence from likely damaging mutation consequences (i.e., null, frameshift, loss of function, missense, gain-of-function, knockout, in-frame, or nonsense mutations) were considered. In addition, as the specific symptoms of affected children were diagnosed (see below for details on measures and clinical evaluations), genes overrepresented at a Benjamini–Hochberg corrected *p* ≤ 0.05 in diseases and disorders reflecting these symptoms were selected for follow-up. These genes were then evaluated in Path Designer to determine if any direct or indirect relationships were observed or predicted downstream of the four genes encoded in the 15q11.2 BP1-BP2 deletion region.

To further evaluate genes with PDVs that may relate to symptoms observed in affected children, these genes were annotated based on evidence of associations with ASD using the 2020 Q2 release of the Simons Foundation for Autism Research Initiative (SFARI) gene list (https://gene.sfari.org/about-gene-scoring/) (accessed on 6 February 2021). In addition, genes were evaluated for inclusion on panels from Fulgent Genetics (https://www.fulgentgenetics.com/) (accessed on 6 February 2021) and Prevention Genetics (https://www.preventiongenetics.com/) (accessed on 6 February 2021), both Clinical Laboratory Improvement Amendments (CLIA) approved and accredited commercial laboratories. Interrogated panels from Fulgent Genetics included: epilepsy, intellectual disability, comprehensive cardiovascular defects, ataxia, microcephaly, macrocephaly, cerebral cortical malformations, neuronal migration disorders and malformations including cleft palate. Genes for connective tissue disorders reflected a combination of both the Fulgent Genetics and Prevention Genetics panels as the latter panel was more comprehensive.

## 5. Conclusions

Our study of the 15q11.2 BP1-BP2 deletion (Burnside-Butler) syndrome, an emerging disorder, found variants in genes beyond the four genes in the chromosome 15q11.2 BP1-BP2 region using exome sequencing with our inclusion criteria and correlations with reported gene and protein interactions with associated diseases or findings. Although we cannot claim that the gene variants found are causal for the clinical, behavioral, or cognitive findings observed (e.g., 9/11 subjects had adaptive functioning abilities below mean for age; 8/11 subjects demonstrated repetitive behavioral scores comparable to ASD subjects; 4 affected children tested had delayed motor milestones; joint instability or hyperextensibility was commonly observed) our data suggests that variants in genes encoded outside of the 15q11.2 BP1-BP2 region may be of interest to future research. Notably, four of the five probands in the five unrelated families inherited the 15q11.2 BP1-BP2 deletion from the mother while only one father (Family E) had the deletion. The affected child in Family E was also the most severely affected (ataxia, wheel-chair user, non-verbal, impaired cognition). This may indicate parent of origin effects, similar to those reported in Davis et al. [57], which included lower motor function and coordination when of paternal origin and more cognitive and behavioral disturbances and seizures when of maternal origin. It is also important to note that several families included in our study traveled long distances. The travel and testing required for participation may have selected for affected children and parents that are relatively less severe than others with this deletion. It is possible that many less severe symptoms reported in this analysis dataset relate primarily to dysfunction in the four core genes encoded in the 15q11.2 BP1-BP2 deleted region. Future studies focused on ascertaining individuals with more severe expression of the symptoms we report may identify more concrete evidence for damaging variants in other genes in addition to the four core genes. Importantly, replication and functional studies are needed to confirm these hypotheses. As noted in our study, a neurodevelopmental phenotype (autism, speech delay, abnormal reflexes, and coordination) was most commonly found along with craniofacial findings (ear anomalies), and orthopedic or connective tissue problems (flat feet, scoliosis, hypermobile joints). These clinical, cognitive, behavioral, and genomic characterizations with reported protein interactions of the four genes of interest and associated diseases in this chromosome 15q11.2 BP1-BP2 deletion did support a role in the neurodevelopmental-autism phenotype seen in this emerging syndrome. Interaction with genes found and their paralogs may have contributed, but our observations are preliminary on a small sample size requiring replication. The authors hope that our findings will stimulate more research and directions for study, shedding light on diagnosis, treatment, and prognosis for affected individuals with this emerging syndrome having the most frequent microarray defect seen in patients presenting with neurodevelopmental disorders.

## Figures and Tables

**Table 1:** Cognitive testing and results for families with 15q11.2 BP1-BP2 deletion.

	Family A			Family B		Family C		Family D		Family E	
Tests	52 yr Mother (Subject1)	15 yr Male (Subject2)	6 yr Male (Subject3)	54 yr Mother (Subject4)	16 yr Female (Subject5)	33 yr Mother (Subject6)	10 yr Male (Subject7)	37 yr Mother (Subject8)	9 yr Female (Subject9)	33 yr Father (Subject10)	6 yr Female (Subject11)
***WASI-II IQ***											
**Perceptual**	104	105	79	142	116	112	89	97	83	92	NT
**Verbal**	96	93	94	111	125	94	99	101	102	109	NT
**Full Scale**	100	99	84	128	124	103	93	99	92	101	NT
***WRAT-IV***											
**Reading**	92	103	75	98	115	95	99	99	112	128	NT
**Math**	96	82	88	124	116	97	72	90	100	105	NT
**Spelling**	96	75	79	125	134	109	85	106	87	102	NT
***CVLT-II/CVLT-C***											
**Short Delay Free Recall**	−1.5	0.5	−1.0	1.0	−0.5	0.5	0.0	1.0	−0.5	−0.5	NT
**Short Delay Cued Recall**	−1.5	0.5	−1.5	1.0	0.5	1.0	−1.0	0.5	−1.5	−0.5	NT
**Long Delay Free Recall**	−1.5	0.0	−2.5	1.0	0.5	1.0	−1.0	0.0	−0.5	−1.5	NT
**Long Delay Cued Recall**	−1.5	0.5	−2.5	1.0	0.5	0.5	−0.5	0.0	−1.0	−0.5	NT
**Recognition**	−2.5	0.5	0.5	0.0	−0.5	0.0	1.5	0.0	1.0	−3	NT
***PPVT-4***											
**Total Score**	99	119	120	107	130	106	107	97	104	104	NT
***Trail Making Test***											
**Part A**	130	129	92	126	99	110	88	111	107	119	NT
**Part B**	126	117	104	75	82	106	71	103	95	104	NT

Age in years (yr) and subject number for family members with the 15q11.2 BP1-BP2 deletion are indicated. NT: Not tested due to non-compliance. Scores for the CVLT are reported as z-scores (mean = 0, std = ±1), scores for all other measures are normed scores with mean = 100, std = ±15.

**Table 2:** Behavioral testing and results for families with 15q11.2 BP1-BP2 deletion syndrome.

	Family A			Family B		Family C		Family D		Family E	
Behavioral Tests	52 yr Mother (Subject1)	15 yr Male (Subject2)	6 yr Male (Subject3)	54 yr Mother (Subject4)	16 yr Female (Subject5)	33 yr Mother (Subject6)	10 yr Male (Subject7)	37 yr Mother (Subject8)	9 yr Female (Subject9)	33 yr Father (Subject10)	6 yr Female (Subject11)
**ADOS-2**											
**RRB**	NT	1	3	NT	0	NT	0	NT	4	NT	3 *
**Social Affect**	NT	11	3	NT	3	NT	4	NT	5	NT	19 *
**Total (ASD Cutoff = 7)**	NT	12	6	NT	3	NT	4	NT	9	NT	22 *
**Classification**	NT	Autism	Non-Autism	NT	Non-Autism	NT	Non-Autism	NT	Autism	NT	Autism
**BAP-Q**											
**Total Score** **(cut-offs: M = 3.55, F = 3.17)**	5.33	NT	NT	1.11	NT	3.67	NT	0.89	NT	3.28	NT
**VABS-III (Standard Scores)**											
**Communication**	NT	54	77	NT	97	NT	70	NT	69	NT	36
**Daily Living**	NT	60	82	NT	98	NT	92	NT	105	NT	46
**Socialization**	NT	40	82	NT	82	NT	80	NT	80	NT	51
**Adaptive Behavior Composite**	NT	55	78	NT	90	NT	59	NT	82	NT	48
**RBS-R**											
**Overall Score**	10	39	9	2	18	9	21	3	15	8	31

Age in years (yr) and subject number for family members with the 15q11.2 BP1-BP2 deletion are indicated. RRB: Restricted and repetitive behaviors, NT: not tested—measure was only administered to either parent or child cohort. * These results are from ADOS-2 Module 1 rather than Module 3.

**Table 3:** Group comparisons of postural ability in children and adults with 15q11.2 BP1-BP2 deletion syndrome and matched controls.

	Children			Adults		
Postural Control Variables	BBS (*n* = 6)	Control (*n* = 6)		BBS (*n* = 5)	Control (*n* = 4)	
	Mean (SD)	Mean (SD)	Cohen’s d	Mean (SD)	Mean (SD)	Cohen’s d
**COP Length (cm)**	35.74 (21.37)	23.81 (15.36)	−0.641	19.45 (11.15)	11.95 (1.36)	−0.944
**ML SD (log)**	−0.49 (0.34)	−0.75 (0.40)	−0.702	−1.02 (0.31)	−1.06 (0.13)	−0.166
**AP SD (log)**	−0.32 (0.31)	−0.42 (0.15)	−0.383	−0.41 (0.32)	−0.57 (0.19)	−0.606

Group means and standard deviations (SD) for children and parents with the 15q11.2 BP1-BP2 deletion (BBS) and their matched controls as well as effect sizes (Cohen’s *d*) for group comparisons. Negative effect sizes indicate greater variability for BBS children or adults relative to controls. COP: center of pressure, ML SD: medial-lateral standard deviation, AP SD: anterior-posterior standard deviation.

**Table 4:** Medical history of families with 15q11.2 BP1-BP2 deletion.

Medical History	Family A			Family B		Family C		Family D		Family E	
	52 yr Mother (Subject1)	15 yr Male (Subject2)	6 yr Male (Subject3)	54 yr Mother (Subject4)	16 yr Female (Subject5)	33 yr Mother (Subject6)	10 yr Male (Subject7)	37 yr Mother (Subject8)	9 yr Female (Subject9)	33 yr Father (Subject10)	6 yr Female (Subject11)
**Prenatal**	Mother age 46 yr			Pre-eclampsia		Gestational diabetes					
**Birth**		C-section	C-section		C-section		C-section				
**Birth weight**		3.4 kg (30th%)	3.6 kg (50th%)				3.7 kg (55th%)				3.8 kg (75th%)
**Neuro-develop-mental**	Learning difficulties	Autism spectrum disorder	Global develop-mental delay				Dyslexia, regression		Learning difficulties		Regression at 12mo
**Neuro-** **psychiatric**	Depression	ADHD, OCD, anxiety			ADHD, GAD, OCD, social phobia, selective mutism		ADHD, OCD, ODD				Sleep disturbance
**Neurological**			Non-essential tremor			Right calf paresthesia	Epilepsy		Hypotonia, fine and gross motor delay		Epilepsy (Lennox-Gastaut syndrome)
**Eye**				Astigmatism					Left strabismus		No retinal folds
**Musculo-** **skeletal**			Leg braces, ankle instability	Herniated disc lumbar 5	Kyphosis, hyper-flexible	Scoliosis		Scoliosis			
**Motor**		Weak postural control, walked at 16 mo	Fine and gross motor delay, walked at 25 mo				Walked at 12 months		Crawled at 20 months, walked at 26 months		Sat at 13mo, crawled at 18mo, poor balance and coordination
**Cardio-** **vascular**	Hyper-tension Hyper-lipidemia				Patent foramen ovale, Postural hypotension					Hyper-tension	
**Respiratory**					Asthma						
**Gastro-** **intestinal**			Consti-pation						Consti-pation		
**Skin**			Easy bruising, delayed healing		Eczema, delayed healing						
**Endocrine**	Diabetes mellitus			Post-partum thyroiditis	Delayed bone age, delayed growth	Gestational diabetes					
**Reproductive**	PCOS										
**Immunologic**				Thymic hyper-trophy	Anaphy-laxis						

Age in years (yr) and subject number for family members with the 15q11.2 BP1-BP2 deletion are indicated. PCOS—polycystic ovarian syndrome, ADHD—attention deficit hyperactivity disorder, OCD—obsessive compulsive disorder, ODD—oppositional defiant disorder, GAD—generalized anxiety disorder.

**Table 5:** Specific clinical evaluation and physical exam findings of families with 15q11.2 BP1-BP2 deletion.

Physical Exam	Family A			Family B		Family C		Family D		Family E	
	52 yr Mother (Subject1)	15 yr Male (Subject2)	6 yr Male (Subject3)	54 yr Mother (Subject4)	16 yr Female (Subject5)	33 yr Mother (Subject6)	10 yr Male (Subject7)	37 yr Mother (Subject8)	9 yr Female (Subject9)	33 yr Father (Subject10)	6 yr Female (Subject11)
**Head circumference**	57.3 cm (75th%)	56.6 cm (75th%)	51.5 cm (50th%)	56.8 cm (90th%)	53.3 cm (25th%)	56.2 cm (50th%)	54.2 cm (85th%)	55.5 cm (50th%)	55.7 cm (>97th%)	57.8 cm (75th%)	49.5 cm (25th%)
**Inner canthal distance**	3.3 cm (75th%)	2.9 cm (50th%)	2.7 cm (50th%)	3.2 cm (75th%)	3.2 cm (75th%)	2.5 cm (3rd%)	3.2 cm (75th%)			3.1 cm (50th%)	3.0 cm (50th%)
**Outer canthal distance**	8.3 cm (25th%)	8.6 cm (50th%)	7.3 cm (3rd%)	8.4 cm (25th%)	8.3 cm (25th%)	8.0 cm (3rd%)	8.4 cm (50th%)			9.2 cm (75th%)	8.7 cm (75th%)
**Right hand length**	19.2 cm (97th%)	20.2 cm (97th%)	15.6 cm (97th%)	16.3 cm (3rd%)	17.0 cm (25th%)	18.2 cm (75th%)	15.6 cm (25th%)	16.6 cm (25th%)	15.8 cm (75th%)	19.0 cm (75th%)	13.3 cm (50th%)
**Right middle finger length**	8.3 cm (75th%)	8.8 cm (97th%)	6.7 cm (97th%)	7.2 cm (3rd%)	7.6 cm (25th%)	7.6 cm (25th%)	6.9 cm (50th%)	7.2 cm (3rd%)	6.8 cm (75th%)	8.1 cm (75th%)	5.2 cm (25th%)
**Right ear length**	7.4 cm (75th%)	6.7 cm (75th%)	6.2 cm (75th%)	6.8 cm (75th%)	6.1 cm (50th%)	6.8 cm (75th%)	6.4 cm (75th%)	6.2 cm (50th%)	5.7 cm (25th%)	NA	5.7 cm (50th%)
**Weight**	110 kg (>95th%)	67.1 kg (80th%)	26.3 kg (90th%)	67.1 kg (75th%)	45.8 kg (10th%)	89.1 kg (>95th%)	32.5 kg (50th%)	79.3 kg (90th%)	35.5 kg (80th%)	123.2 kg (>95th%)	27.6 kg (>95th%)
**Height**	176 cm (>95th%)	182 cm (95th%)	137 cm (>95th%)	158 cm (20th%)	158 cm (20th%)	158.5 cm (20th%)	139 cm (50th%)	159 cm (25th%)	142 cm (90th%)	178 cm (50th%)	NA
**BMI**	35.5 (>95th%)	24.9 (90th%)	14.0 (5th%)	26.9 (85th%)	18.3 (20th%)	35.5 (>95th%)	16.8 (50th%)	31.4 (95th%)	17.6 (70th%)	38.9 (>95th%)	NA
**Head/Facial features**		Soft ears, fold easily	Broad, soft ears	Small upper incisor (#10)	Small upper incisor (#10)		Flat occiput, fleshy ears, thin upper lip, and flat philtrum		Right ear overfolded	Broad, round face	Broad, round face, broad nose, full lips, prominent jaw, forehead, and ears, depressed nasal bridge
**Eyes**		Pigment changes, Sensitive to light	Pigment changes, Sensitive to light		Left pupil larger than right	Mild left ptosis	Mild bilateral ptosis (L > R)			Myopia, right sided ptosis	
**Back**	No scoliosis	No scoliosis	Right shoulder higher than left, no scoliosis	Mild scoliosis	Kyphosis, right shoulder droop			Mild scoliosis			
**Upper extremities**			Hyper-extensibility: Beighton score 6/9		Hyper-extensibility: Beighton score 7/9, cubitus valgus					Broad hands	Soft fleshy, broad hands
**Lower extremities**			Second toes overlap third, plantar creases, flat feet, ankle instability on pronation	Left leg longer than right, knee-buttock asymmetry	Knee-buttock asymmetry, flat feet		Left leg longer than right	Flat feet	Flat feet		
**Other musculo-** **skeletal**							Pectus carinatum				Large body size
**Skin**	Birth mark on thigh	Soft, fleshy	Loose, soft, velvety	Eczema	Soft with freckles		Soft				Birth marks on thigh and forehead
**Neurological**					Increased deep tendon reflexes	Decreased sensation right lateral calf		Decreased deep tendon reflexes	Decreased muscle tone and reflexes in all extremities		Toe walking, poor coordination and balance

Age in years (yr) and subject number for family members with the 15q11.2 BP1-BP2 deletion are indicated.

## Data Availability

Genomic data that support the findings of this study are available in the Supplemental Table S1 and additional data are available from the authors upon reasonable request.

## Supplementary Materials

The following are available online at https://www.mdpi.com/1422-0067/22/4/1660/s1.

## References

[1] Nicholls R.D., Knoll J.H., Butler M.G., Karam S., Lalande M. (1989). Genetic imprinting suggested by maternal heterodisomy in nondeletion Prader-Willi syndrome. Nature.

[2] Butler M.G., Manzardo A.M., Forster J.L. (2016). Prader-Willi Syndrome: Clinical Genetics and Diagnostic Aspects with Treatment Approaches. Curr. Pediatr. Rev..

[3] Butler M., Lee P.D.K., Whitman B. (2006). Management of Prader-Willi syndrome. Management of Prader-Willi Syndrome.

[4] Williams C.A., Driscoll D.J., Dagli A.I. (2010). Clinical and genetic aspects of Angelman syndrome. Genet. Med..

[5] Bittel D.C., Butler M.G. (2005). Prader-Willi syndrome: Clinical genetics, cytogenetics and molecular biology. Expert Rev. Mol. Med..

[6] Zarcone J., Napolitano D., Peterson C., Breidbord J., Ferraioli S., Caruso-Anderson M., Holsen L., Butler M.G., Thompson T. (2007). The relationship between compulsive behaviour and academic achievement across the three genetic subtypes of Prader-Willi syndrome. J. Intellect. Disabil. Res..

[7] Butler M.G., Bittel D.C., Kibiryeva N., Talebizadeh Z., Thompson T. (2004). Behavioral differences among subjects with Prader-Willi syndrome and type I or type II deletion and maternal disomy. Pediatrics.

[8] Hartley S.L., Maclean W.E., Butler M.G., Zarcone J., Thompson T. (2005). Maladaptive behaviors and risk factors among the genetic subtypes of Prader-Willi syndrome. Am. J. Med. Genet. A.

[9] Burnside R.D., Pasion R., Mikhail F.M., Carroll A.J., Robin N.H., Youngs E.L., Gadi I.K., Keitges E., Jaswaney V.L., Papenhausen P.R. (2011). Microdeletion/microduplication of proximal 15q11.2 between BP1 and BP2: A susceptibility region for neurological dysfunction including developmental and language delay. Hum. Genet..

[10] Cox D.M., Butler M.G. (2015). The 15q11.2 BP1-BP2 microdeletion syndrome: A review. Int. J. Mol. Sci..

[11] Butler M.G. (2017). Clinical and genetic aspects of the 15q11.2 BP1-BP2 microdeletion disorder. J. Intellect. Disabil. Res..

[12] Ho K.S., Wassman E.R., Baxter A.L., Hensel C.H., Martin M.M., Prasad A., Twede H., Vanzo R.J., Butler M.G. (2016). Chromosomal Microarray Analysis of Consecutive Individuals with Autism Spectrum Disorders Using an Ultra-High Resolution Chromosomal Microarray Optimized for Neurodevelopmental Disorders. Int. J. Mol. Sci..

[13] Rafi S.K., Butler M.G. (2020). The 15q11.2 BP1-BP2 Microdeletion (Burnside–Butler) Syndrome: In Silico Analyses of the Four Coding Genes Reveal Functional Associations with Neurodevelopmental Disorders. Int. J. Mol. Sci..

[14] Delis D.C., Kramer J.H., Kaplan E., Ober B.A. (2000). California Verbal Learning Test.

[15] Dunn L.M., Dunn D.M. (2007). Peabody Picture Vocabulary Test.

[16] Reitan R.M. (1958). Validity of the Trail Making Test as an Indicator of Organic Brain Damage. Percept. Mot. Skills.

[17] Gotham K., Pickles A., Lord C. (2009). Standardizing ADOS scores for a measure of severity in autism spectrum disorders. J. Autism Dev. Disord..

[18] Lord C., Luyster R., Guthrie W., Pickles A. (2012). Patterns of developmental trajectories in toddlers with autism spectrum disorder. J. Consult. Clin. Psychol..

[19] Sparrow S.S., Ciccheti D.V., Saulnier C.A. (2016). Vineland Adaptive Behavior Scales.

[20] Esbensen A.J., Seltzer M.M., Lam K.S., Bodfish J.W. (2009). Age-related differences in restricted repetitive behaviors in autism spectrum disorders. J. Autism Dev. Disord..

[21] Hurley R.S., Losh M., Parlier M., Reznick J.S., Piven J. (2007). The broad autism phenotype questionnaire. J. Autism Dev. Disord..

[22] Sasson N.J., Lam K.S.L., Childress D., Parlier M., Daniels J.L., Piven J. (2013). The broad autism phenotype questionnaire: Prevalence and diagnostic classification. Autism Res..

[23] Bodfish J.W., Symons F.J., Parker D.E., Lewis M.H. (2000). Varieties of repetitive behavior in autism: Comparisons to mental retardation. J. Autism Dev. Disord..

[24] Quamme G.A. (2010). Molecular identification of ancient and modern mammalian magnesium transporters. Am. J. Physiol. Cell Physiol..

[25] Rainier S., Chai J.-H., Tokarz D., Nicholls R.D., Fink J.K. (2003). NIPA1 Gene Mutations Cause Autosomal Dominant Hereditary Spastic Paraplegia (SPG6). Am. J. Hum. Genet..

[26] Maenner M.J. (2020). Prevalence of Autism Spectrum Disorder Among Children Aged 8 Years—Autism and Developmental Disabilities Monitoring Network, 11 Sites, United States, 2016. MMWR Surv. Summ..

[27] Capodaglio P., Menegoni F., Vismara L., Cimolin V., Grugni G., Galli M. (2011). Characterisation of balance capacity in Prader-Willi patients. Res. Dev. Disabil..

[28] Wang Z., Hallac R.R., Conroy K.C., White S.P., Kane A.A., Collinsworth A.L., Sweeney J.A., Mosconi M.W. (2016). Postural orientation and equilibrium processes associated with increased postural sway in autism spectrum disorder (ASD). J. Neurodev. Disord..

[29] Lim Y.H., Partridge K., Girdler S., Morris S.L. (2017). Standing Postural Control in Individuals with Autism Spectrum Disorder: Systematic Review and Meta-analysis. J. Autism Dev. Disord.

[30] Wang Z., Khemani P., Schmitt L.M., Lui S., Mosconi M.W. (2019). Static and dynamic postural control deficits in aging fragile X mental retardation 1 (FMR1) gene premutation carriers. J. Neurodev. Disord..

[31] Errico A., Ballabio A., Rugarli E.I. (2002). Spastin, the protein mutated in autosomal dominant hereditary spastic paraplegia, is involved in microtubule dynamics. Hum. Mol. Genet..

[32] Evans K.J., Gomes E.R., Reisenweber S.M., Gundersen G.G., Lauring B.P. (2005). Linking axonal degeneration to microtubule remodeling by Spastin-mediated microtubule severing. J. Cell Biol..

[33] Alber B., Pernauer M., Schwan A., Rothmund G., Hoffmann K.T., Brummer D., Sperfeld A.D., Uttner I., Binder H., Epplen J.T. (2005). Spastin related hereditary spastic paraplegia with dysplastic corpus callosum. J. Neurol. Sci..

[34] Munhoz R.P., Kawarai T., Teive H.A., Raskin S., Sato C., Liang Y., St George-Hyslop P.H., Rogaeva E. (2006). Clinical and genetic study of a Brazilian family with spastic paraplegia (SPG6 locus). Mov. Disord..

[35] Matthews A.M., Tarailo-Graovac M., Price E.M., Blydt-Hansen I., Ghani A., Drögemöller B.I., Robinson W.P., Ross C.J., Wasserman W.W., Siden H. (2017). A de novo mosaic mutation in SPAST with two novel alternative alleles and chromosomal copy number variant in a boy with spastic paraplegia and autism spectrum disorder. Eur. J. Med. Genet..

[36] Henkhaus R.S., Kim S.J., Kimonis V.E., Gold J.A., Dykens E.M., Driscoll D.J., Butler M.G. (2012). Methylation-specific multiplex ligation-dependent probe amplification and identification of deletion genetic subtypes in Prader-Willi syndrome. Genet. Test Mol. Biomark..

[37] Liu C., Mei M., Li Q., Roboti P., Pang Q., Ying Z., Gao F., Lowe M., Bao S. (2017). Loss of the golgin GM130 causes Golgi disruption, Purkinje neuron loss, and ataxia in mice. Proc. Natl. Acad. Sci. USA.

[38] Matsuki T., Matthews R.T., Cooper J.A., van der Brug M.P., Cookson M.R., Hardy J.A., Olson E.C., Howell B.W. (2010). Reelin and stk25 have opposing roles in neuronal polarization and dendritic Golgi deployment. Cell.

[39] Futreal P.A., Cochran C., Rosenthal J., Miki Y., Swenson J., Hobbs M., Bennett L.M., Haugen-Strano A., Marks J., Barrett J.C. (1994). Isolation of a diverged homeobox gene, MOX1, from the BRCA1 region on 17q21 by solution hybrid capture. Hum. Mol. Genet..

[40] Mankoo B.S., Skuntz S., Harrigan I., Grigorieva E., Candia A., Wright C.V., Arnheiter H., Pachnis V. (2003). The concerted action of Meox homeobox genes is required upstream of genetic pathways essential for the formation, patterning and differentiation of somites. Development.

[41] Ricard-Blum S. (2011). The collagen family. Cold Spring Harb. Perspect. Biol..

[42] Fitzgerald J., Holden P., Hansen U. (2013). The expanded collagen VI family: New chains and new questions. Connect. Tissue Res..

[43] Mukhopadhyay A., Kramer J.M., Merkx G., Lugtenberg D., Smeets D.F., Oortveld M.A., Blokland E.A., Agrawal J., Schenck A., van Bokhoven H. (2010). CDK19 is disrupted in a female patient with bilateral congenital retinal folds, microcephaly and mild mental retardation. Hum. Genet..

[44] Wechsler D., Zhou X. (2011). WASI-II: Wechsler Abbreviated Scale of Intelligence.

[45] Wilkinson G.S., Robertson G.J. (2006). WRAT 4: Wide Range Achievement Test.

[46] Cohen J. (1988). Statistical Power Analysis for the Behavioral Sciences.

[47] Andrews S. FastQC: A Quality Control Tool for High Throughput Sequence Data. http://www.bioinformatics.babraham.ac.uk/projects/fastqc2010.

[48] Li H., Durbin R. (2009). Fast and accurate short read alignment with Burrows-Wheeler transform. Bioinformatics.

[49] Van der Auwera G.A., Carneiro M.O., Hartl C., Poplin R., Del Angel G., Levy-Moonshine A., Jordan T., Shakir K., Roazen D., Thibault J. (2013). From FastQ data to high confidence variant calls: The Genome Analysis Toolkit best practices pipeline. Curr. Protoc. Bioinform..

[50] Cingolani P., Platts A., Wang L.L., Coon M., Nguyen T., Wang L., Land S.J., Lu X., Ruden D.M. (2012). A program for annotating and predicting the effects of single nucleotide polymorphisms, SnpEff: SNPs in the genome of Drosophila melanogaster strain w1118; iso-2; iso-3. Fly.

[51] McLaren W., Gil L., Hunt S.E., Riat H.S., Ritchie G.R., Thormann A., Flicek P., Cunningham F. (2016). The Ensembl Variant Effect Predictor. Genome Biol..

[52] Ng P.C., Henikoff S. (2003). SIFT: Predicting amino acid changes that affect protein function. Nucleic Acids Res..

[53] Adzhubei I., Jordan D.M., Sunyaev S.R. (2013). Predicting functional effect of human missense mutations using PolyPhen-2. Curr. Protoc. Hum. Genet..

[54] Grantham R. (1974). Amino acid difference formula to help explain protein evolution. Science.

[55] Krämer A., Green J., Pollard J., Tugendreich S. (2014). Causal analysis approaches in Ingenuity Pathway Analysis. Bioinformatics.

[56] Wang W., Corominas R., Lin G.N. (2019). De novo Mutations From Whole Exome Sequencing in Neurodevelopmental and Psychiatric Disorders: From Discovery to Application. Front. Genet..

[57] Davis K.W., Serrano M., Loddo S., Robinson C., Alesi V., Dallapiccola B., Novelli A., Butler M.G. (2019). Parent-of-Origin Effects in 15q11.2 BP1-BP2 Microdeletion (Burnside-Butler) Syndrome. Int. J. Mol. Sci..

